# Immunosenescence in Childhood Cancer Survivors and in Elderly: A Comparison and Implication for Risk Stratification

**DOI:** 10.3389/fragi.2021.708788

**Published:** 2021-07-19

**Authors:** Petra Lázničková, Kamila Bendíčková, Tomáš Kepák, Jan Frič

**Affiliations:** ^1^ International Clinical Research Center, St. Anne’s University Hospital Brno, Brno, Czech Republic; ^2^ Department of Biology, Faculty of Medicine, Masaryk University, Brno, Czech Republic; ^3^ Department of Pediatric Oncology, University Hospital Brno, Brno, Czech Republic; ^4^ Institute of Hematology and Blood Transfusion, Prague, Czech Republic

**Keywords:** childhood cancer survivor, immunosenescence, low-grade inflammation, accelerated aging, late effects, patient stratification, elderly

## Abstract

The population of childhood cancer survivors (CCS) has grown rapidly in recent decades. Although cured of their original malignancy, these individuals are at increased risk of serious late effects, including age-associated complications. An impaired immune system has been linked to the emergence of these conditions in the elderly and CCS, likely due to senescent immune cell phenotypes accompanied by low-grade inflammation, which in the elderly is known as “inflammaging.” Whether these observations in the elderly and CCS are underpinned by similar mechanisms is unclear. If so, existing knowledge on immunosenescent phenotypes and inflammaging might potentially serve to benefit CCS. We summarize recent findings on the immune changes in CCS and the elderly, and highlight the similarities and identify areas for future research. Improving our understanding of the underlying mechanisms and immunosenescent markers of accelerated immune aging might help us to identify individuals at increased risk of serious health complications.

## Introduction

**FIGURE 1:**
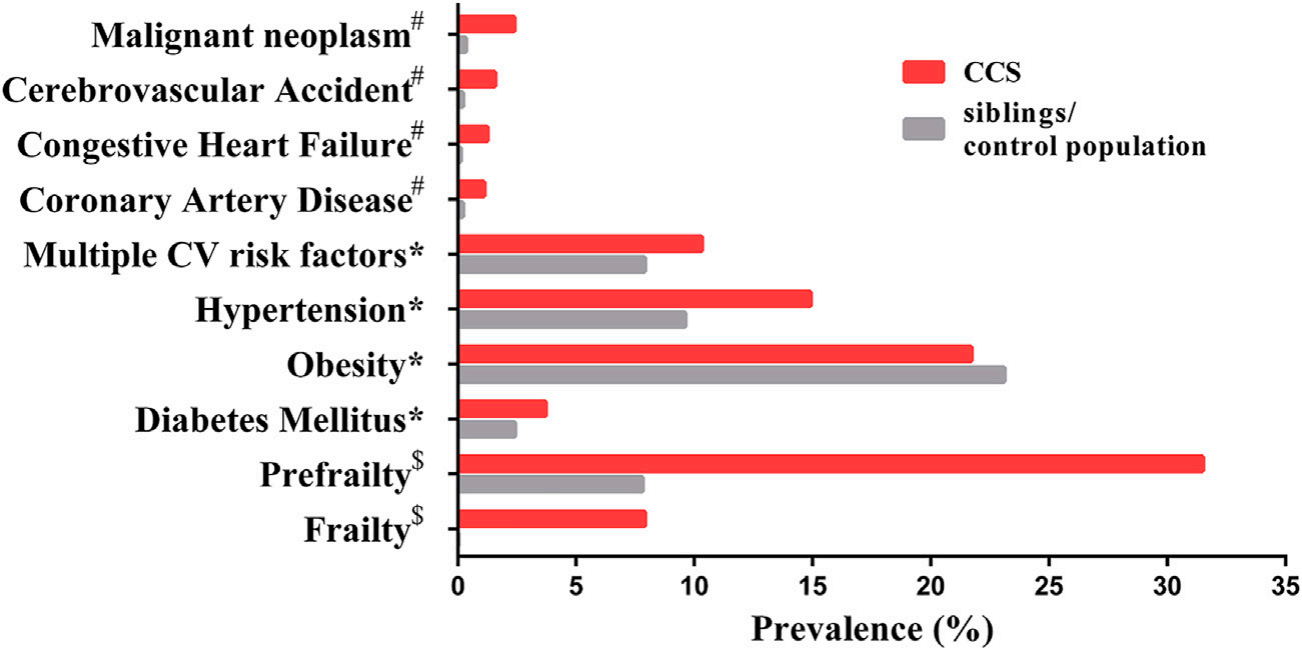
The prevalence of age-associated complications in CCS and siblings/control population. Data marked ^#^ has a mean age of 26.6 years (range 18–48) for CCS and a mean age of 29.2 years (range 18–56) for siblings (Oeffinger et al., 2006); data marked * has a median age of 33.7 years (range 11–58) in CCS and a median age of 36 years (range 7–62) for siblings (Armstrong et al., 2013); data marked ^$^ has a mean age of 33.6 years (range 18–50) for CCS and a mean age of 29 years (range 18–50) for the control population (Ness et al., 2013). Malignant neoplasm represents secondary neoplasms for CCS and primary neoplasms for siblings/control population. Abbreviation: CV, cardiovascular.

Each year, ∼300,000 children are diagnosed with a form of cancer globally. (Steliarova-Foucher et al., 2017). Cancer therapy has made immense progress in recent decades, increasing the 5 year survival rate to up to 85% in countries with advanced healthcare systems (Gatta et al., 2014; Howlader et al., 2020). Yet with this great step forward, a significant problem has emerged: childhood cancer survivors (CCS) have a higher incidence of developing other, severe health conditions compared to their siblings (Armstrong et al., 2014), with up to 75% experiencing at least one late adverse effect and 40% suffering from at least one serious or life-threatening condition in early-mid adulthood (Geenen et al., 2007). The earlier prevalence of certain health complications in CCS compared to their siblings (or control population) (Figure 1) is a phenomenon known as premature aging. These complications include cardiovascular diseases, frailty, and secondary neoplasms (Oeffinger et al., 2006; Armstrong et al., 2013; Ness et al., 2013). Frailty is described by experiencing three or more conditions of established frailty phenotype (low lean muscle mass, exhaustion, low energy expenditure, slowness and weakness), while prefrailty is described by experiencing two of these conditions conditions (Ness et al., 2013). The early onset of health complications for CCS seems to prove the fragile health status of CCS being more similar to elderly (Table 1). The most pronounced comorbidities in CCS span through many tissues and organ systems, such as pulmonary, cardiac and circulatory, genitourinary, nervous and endocrine (Geenen et al., 2007; Reulen et al., 2010; Armstrong et al., 2014; Bhakta et al., 2017). The diseases developing in above mentioned organ systems are accompanied by metabolic changes, higher rate of infections and subsequent malignancy (Reulen et al., 2010; Lorenzi et al., 2011). In CCS, secondary cancer has been described as the most severe comorbid condition compared to cardiovascular or respiratory diseases (Reulen et al., 2010). The emergence of these conditions is thought to be driven by late effects of the often aggressive treatments needed to cure the childhood cancer, (Gallicchio et al., 2008; Fowler et al., 2020), but the precise mechanisms are unknown. However, cancer therapeutics have been already associated with cellular aging through senescence initiation, free radical generation, DNA damage and telomere attrition (Cupit-Link et al., 2017; Wang et al., 2021).

**TABLE 1:** Adverse health conditions associated with accelerated senescence in CCS and the elderly.

Comorbidities	CCS	Healthy elderly
Frailty	Ness et al. (2013), Vatanen et al. (2017)	Fried et al. (2001)
Infections	Reulen et al. (2010), Lorenzi et al. (2011), Perkins et al. (2014)	Gavazzi et al. (2002)
Blood diseases	Reulen et al. (2010), Lorenzi et al. (2011), Armstrong et al. (2014)	Wolff et al. (2002)
Reduced microbial diversity	Chua et al. (2017)	Woodmansey et al. (2004), Xu et al. (2019)
Obesity and diabetes mellitus	Mostoufi-Moab et al. (2016)	Marengoni et al. (2009), Liu et al. (2018)
Metabolic changes	Chow et al. (2010), Lorenzi et al. (2011), Azanan et al. (2016), Ariffin et al. (2017), Vatanen et al. (2017), Daiel et al. (2018)	Wolff et al. (2002), Liu et al. (2018)
Pulmonary disease	Mertens et al. (2008), Reulen et al. (2010), Armstrong et al. (2014), Bhakta et al. (2017)	Wolff et al. (2002), Marengoni et al. (2009)
Cardiac and circulatory disease	Mertens et al. (2008), Reulen et al. (2010), Armstrong et al. (2014), Bhakta et al. (2017), Bates et al. (2019), Effinger et al. (2019)	Wolff et al. (2002), Marengoni et al. (2009), Liu et al. (2018)
Genitourinary disease	Geenen et al. (2007), Reulen et al. (2010), Felicetti et al. (2011), Brignardello et al. (2013)	Wolff et al. (2002)
Nervous system disease, stroke	Geenen et al. (2007), Reulen et al. (2010), Lorenzi et al. (2011), Armstrong et al. (2014)	Wolff et al. (2002), Marengoni et al. (2009)
Musculoskeletal disorders	Geenen et al. (2007), Armstrong et al. (2014), Bhakta et al. (2017)	Wolff et al. (2002), Marengoni et al. (2009)
Endocrine disease, Growth hormone deficiency	Geenen et al. (2007), Reulen et al. (2010), Felicetti et al. (2011) Lorenzi et al. (2011), Brignardello et al. (2013), Armstrong et al. (2014), Mostoufi-Moab et al. (2016), Bhakta et al. (2017)	Wolff et al. (2002)
Subsequent malignancy, secondary cancer	Geenen et al. (2007), Mertens et al. (2008), Reulen et al. (2010), Lorenzi et al. (2011), Armstrong et al. (2014), Mostoufi-Moab et al. (2016), Bhakta et al. (2017), Effinger et al. (2019)	Marengoni et al. (2009)
Epigenetic changes	Daniel et al. (2018)	Bollati et al. (2009), Christensen et al. (2009)

Many conditions affecting CCS in early/mid-adulthood are age-associated; cardiovascular diseases, diabetes mellitus, hypertension, frailty and cancer increase during aging (Partridge et al., 2018). Aging itself is a complex process that ultimately results in the gradual decline of various critical cellular processes, signaling pathways, and regulatory mechanisms, leading to eventual disruption of tissue homeostasis and the emergence of disease (López-Otín et al., 2013). The immune compartment is widely affected by aging through the process of progressive dysregulation of immune function. Alterations to the innate and adaptive immune compartments and aging-associated chronic low-grade inflammation (CLGI), (Solana et al., 2012; Fulop et al., 2013), termed “inflammaging” (Franceschi et al., 2000a), are common. Together, these changes are termed “immunosenescence” and contribute to the emergence of various health conditions, such as frailty, (Pansarasa et al., 2019), type 2 diabetes mellitus (Lee et al., 2019), pulmonary diseases (Murray and Chotirmall, 2015), increased susceptibility to infections, autoimmune disease, and cancer (Pawelec, 1999) in aged individuals. Unsurprisingly, strategies aimed at improving aspects of immunosenescence in the elderly are being actively explored.

The immune systems of young adult CCS exhibit common features with those of the elderly (Azanan et al., 2016; Ariffin et al., 2017). High prevalence of typical aging-associated conditions in CCS suggest that CCS might exhibit premature immunosenescence as a result of their cancer and/or its treatment. Understanding this phenomenon will help determine whether screening for early immunosenescence will enable the risk-stratification of CCS after treatment and thereby facilitate optimal clinical management, e.g. earlier screening for secondary cancer. Furthermore, if conserved mechanisms are at play in both CCS and elderly patients, emerging therapies to ameliorate immunosenescence in the elderly should be assessed for their potential benefit to CCS.

In this review, we discuss the possible adverse effects of childhood cancer treatment on the immune system and the potential links to enduring ill health in CCS. We compare these effects to the senescent immune system in the “healthy” elderly, assessing the evidence for parallel versus distinct mechanisms in these two populations. We conclude by speculating on how we might exploit our knowledge of cellular, molecular and epigenetic mechanisms of immunosenescence to improve health and wellbeing in CCS and we highlight possible directions for future research in the field.

## Immune Cell Subsets Affected by Aging and Childhood Cancer Treatment

Many of the initial studies defining immunosenescence were performed in unique cohorts of centenarians, allowing the identification of immunosenescent cellular phenotypes/markers that enable successful aging (Effros et al., 1994; Fagnoni et al., 1996; Ostan et al., 2008). These descriptive studies revealed characteristic frequencies of immunosenescence-associated cell subsets (naïve, memory and terminally differentiated T cells) that are now widely used to describe the immunosenescent phenotype in humans. Stemming from this work, several possible mechanisms were proposed to account for the development of immunosenescence, including 1) decrease in naïve T cells accompanied by expansion of memory T cell subsets with age; (Saule et al., 2006) 2) changes in myeloid cells, particularly monocytes; (Hearps et al., 2012) 3) CLGI; (Fülöp et al., 2019) and 4) chronic infection with pathogens such as cytomegalovirus (CMV) (Olsson et al., 2000; Koch et al., 2007; Kallemeijn et al., 2017).

Immune changes in both the elderly and CCS have now been identified (Table 2); below we consider each immune cell subset in turn, identifying the parallels and differences between the two population groups.

**TABLE 2:** The phenotypes of cellular subsets associated with senescence/immunosenescence in CCS and the healthy elderly.

Cell type	Immune cell phenotype	CCS	Healthy elderly
T cells (CD3^+^)	CD4^+^CD38^+^ HLA-DR^+^	ALL, AML (26)	Azanan et al. (2016)
	CD4^+^ central memory	ALL, Hodgkin lymphoma, Non-Hodgkin lymphoma Sulicka-Grodzicka et al. (2021)	Saule et al. (2006)
	CD4^+^CD28^−^	ALL, AML Azanan et al. (2016)	Vallejo et al. (2000), Suarez-Álvarez et al. (2017)
	CD8^+^CD38^+^ HLA-DR^+^	ALL, AML Azanan et al. (2016)	Not found
	CD8^+^ central memory	ALL, Hodgkin lymphoma, Non-Hodgkin lymphoma Sulicka-Grodzicka et al. (2021)	Saule et al. (2006)
	CD8^+^CD28^−^	Not found	Fagnoni et al. (1996), Weng et al. (2009)
Monocytes	CD14^+^CD16^+^	ALL Sulicka et al. (2013)	Seidler et al. (2010), Hearps et al. (2012), Ong et al. (2018)
NK cells	CD56^dim^CD57^+^	Not found	Le Garff-Tavernier et al. (2010)

To compare the immunosenescent phenotype among two independent groups, we reviewed cell phenotypes that were marked in the literature as significantly changed in CCS compared to age-matched peers. Abbreviations: ALL, acute lymphoblastic leukemia; AML, acute myeloid leukemia; NK, natural killer cells.

### Adaptive Immune Cells

#### T Cells

**FIGURE 2:**
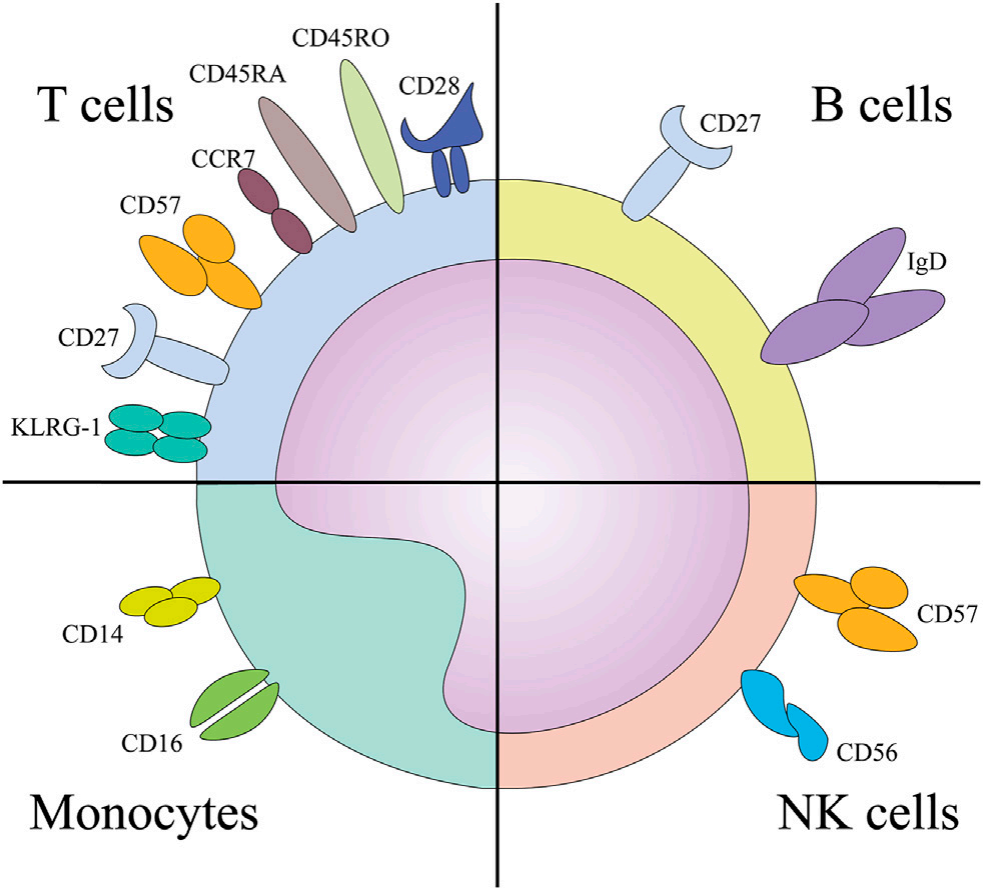
The surface markers implicated in the immunosenescence phenotype of designated cells. Abbreviations: KLRG-1, Killer cell lectin like receptor sub family G member 1; NK, natural killer cells.

Age-associated changes in the subpopulation frequencies of CD4^+^ and CD8^+^ T cells are well established (Di Mitri et al., 2011; Montecino-Rodriguez et al., 2013; Lanna et al., 2014; Callender et al., 2018). Multi-parameter flow cytometry of circulating T-cell subsets in cohorts of elderly individuals has identified increasing proportions of specific T-cell subsets as major immunosenescence markers. The four main T-cell subsets are distinguished based on the recognition of antigens and are delineated as naive (CD45RA^+^, CD45RO^−^, CD27^+^, CD28^+^, and CCR7^+^), central memory (CD45RA^−^, CD45RO^+^, CD27^+^, CD28^+^, and CCR7^+^), effector memory (CD45RA^−^, CD45RO^+^, CD27^−^, CD28^−^, and CCR7^−^) and terminal effector T cells (CD45RA^+^, CD45RO^−^, CD27^−^, CD28^−^ and CCR7^−^) (Wang et al., 1995; Boucher et al., 1998; Hendriks et al., 2000; Larbi and Fulop, 2014; Xu and Larbi, 2017). In general, a decrease in naive T cells and an increase in effector memory and terminal effector T cells during normal aging have been established (Saule et al., 2006). Interestingly, even though markers used to determine immunosenescent phenotype are established (Figure 2), T-cell phenotyping strategies using flow cytometry still vary among human studies. (Fagnoni et al., 1996; Kovaiou et al., 2005; Koch et al., 2008; Lázničková et al., 2020).

The marker expression patterns of senescent T cells are consistent with their altered functionality. Instead of expressing co-stimulatory molecules such as CD27 and CD28, (Hendriks et al., 2000; Weng et al., 2009; Parish et al., 2010) senescent T cells express Killer cell lectin-like receptor subfamily G member 1 (KLRG-1) and CD57, (Brenchley et al., 2003; Wherry, 2011) which are associated with low proliferative capacity but high cytotoxic potential (Ibegbu et al., 2005; Kared et al., 2016). Furthermore, these cells exhibit elevated reactive oxygen species (ROS) production and constitutive p38 MAP kinase activation in response to AMP-activated protein kinase (AMPK) and possibly a nutrient-sensing and/or DNA-damage response (Lanna et al., 2014; Akbar et al., 2016). Senescent T cells are also characterized by shorter telomeres, the inability to upregulate telomerase expression, inadequate expression of the DNA damage response machinery, increased cyclin-dependent kinase inhibitor p16^INK4a^ expression, and elevated secretion of inflammatory cytokines (Akbar et al., 2016). Chemotherapy regimens and stem cell transplantation cause T cell senescence through p16INK4a expression in patients treated for hematological malignancy (Wood et al., 2016) and chemotherapy can induce surface expression of CD57 in Vδ2pos T cells as demonstrated in elderly patients with liver metastatic colorectal cancer (Bruni et al., 2019). On top of that, it has been shown that T cells from patients with hematological malignancy show higher clonality and lower T cell diversity based on T cell receptor repertoire analysis by next-generation sequencing than those with solid tumors, when compared to healthy donors above 60 yo (Simnica et al., 2019). Therefore, certain therapeutic approaches may represent a risk for senescence induction in T cells with the potential to compromise the overall health status of cancer survivors. Moreover, a mouse study has uncovered that senescence driven by Ercc1 deletion (encoding a crucial DNA repair protein) in hematopoietic cells drives senescence also in other non-lymphoid organs, e.g. aorta, heart, lung, liver and gastrointestinal tract, and splenocytes from these mice drive senescence in young senescence reporter mice upon transplant (Yousefzadeh et al., 2021).

T-cell frequency studies in CCS are scarce (Table 2), but we recently showed that high-risk neuroblastoma CCS exhibit immunosenescent-like alterations, including a decrease in naive T-cell frequency and an increase in the frequency of memory T cells, accompanied by the transient expression of CD57 in memory T-cell populations within the first 4 years after diagnosis (Lázničková et al., 2020). Other T-cell phenotyping studies in CCS have also detected the loss of CD28 and increase in CD57 in CD4^+^ T cells (Azanan et al., 2016). The studies of activation status of T cells in CCS and elderly are unparalleled. Increased activation of CD8^+^ T cells by double positivity for CD38 and HLA-DR has been detected in CCS, (Azanan et al., 2016) while the activation status by only CD38 increased (Sulicka-Grodzicka et al., 2021) and by HLA-DR only decreased in CD8^+^ T cells in CCS (Chua et al., 2017). In the elderly, the activation status by only CD38 has been found to be decreased and by only HLA-DR increased in CD8^+^ T cells in elderly (Qin et al., 2016). Interestingly, terminally differentiated CD8^+^ T cells do not show a senescent phenotype in CCS like in elderly, while the central memory CD8^+^ and CD4^+^ T cells increase in both, CCS and elderly (Saule et al., 2006; Sulicka-Grodzicka et al., 2021).

In addition to phenotyping and telomere length analyses, epigenetic remodeling in blood cells contributes to the alterations in genome that accompany aging (Weidner et al., 2014). By this approach, researchers have identified aging-related T-cell epigenetic remodeling in CCS (Daniel et al., 2018). For example, total body irradiation alters the DNA methylation signature of T cells, which has been associated with an increased frequency of type 1 cytokine-producing T cells and increased systemic levels of these cytokines (Daniel et al., 2018). Epigenetic changes might, therefore, explain why radiotherapy confers up to an eight-fold increased risk of a severe or life-threatening medical condition (Oeffinger et al., 2006; Armstrong et al., 2010; Ness et al., 2018).

#### B Cells

The role of B cells in immunosenescence is relatively under-studied and thus their contribution to this process is elusive. In the peripheral blood, four major B-cell subsets can be distinguished, based on expression patterns of surface molecules: naive (IgD^+^/CD27^−^), IgM memory (IgD^+^/CD27^+^), switched memory (IgD^−^/CD27^+^), and late/exhausted memory (LM, IgD^−^/CD27^−^). While total B-cell counts remain relatively unchanged in the peripheral blood in healthy adults, (Blanco et al., 2018) recent findings have indicated a shift in the B-cell subset distribution in the elderly in favor of LM B cells. This shift is accompanied by increased expression of senescence-associated secretory phenotype (SASP) markers, including TNF-α, IL-6, and IL-8 (Frasca et al., 2017a; Frasca et al., 2017b). Quantification of the LM B-cells subset showed a decrease in CSS and naive B cells an increase in CCS compared to age-matched controls, (Sulicka-Grodzicka et al., 2021) showing a deregulation in the B cell compartment in both directions, to healthy peers and to elderly. The accumulation of the LM B-cell subset contributes to lower vaccine protection against influenza in the elderly and is further decreased in obese individuals (Frasca et al., 2016). CCS are often in need of boosters or complete revaccination after cancer therapy due to active treatment in early childhood resulting in incomplete series of vaccinations and no/low immune recovery depending on the type of implemented treatment (Patel et al., 2007; Shetty and Winter, 2012; Han et al., 2018; Choi et al., 2020). In contrast to children with active chemotherapy treatment, patients who had completed chemotherapy and healthy children had similar, stronger vaccination response efficacies (Goossen et al., 2013). Therefore, monitoring of B cell subsets might be an indicator of the B cell ability to cope with infections and eventually infection rate in general but the subset distribution in CCS does not seem to reflect the aging phenotype described in elderly.

In the year 2011, a subset of B cells designated age-associated B cells (ABCs) was shown to accumulate in aged mice (Hao et al., 2011; Rubtsov et al., 2011). These cells are responsive to Toll-like receptor (TLR) 7 and 9 stimulation, actively secrete IL-10 and IL-4, and favor T-cell polarization towards T helper 17 (Th17) cells. In 2019, their possible counterparts have been identified in humans in relation to autoimmune diseases and viral infections (Ma et al., 2019). Whether these B cells also accumulate during normal aging or in CCS is unclear. Therefore, there is a need of verifying this B cell subset in elderly and find the comorbidities associated with this phenotype. The mouse studies (Hao et al., 2011; Rubtsov et al., 2011) imply that comorbidities linked to higher emergence of Th17 cells (e.g. obesity) might be linked with these ABCs (Frasca et al., 2017a; Blanco et al., 2018). Whether ABCs will be applicable to CCS remains elusive but due to the prevalence of comorbidities similar to those in the elderly, chances are the phenotype might be present.

### Innate Immune Cells

The profound age-associated changes in the immune system underlying immunosenescence are mainly linked to adaptive immunity. However, research conducted over the past two decades has demonstrated many functional age-related alterations in innate immune cells (Shaw et al., 2013; Pinti et al., 2016; Agrawal et al., 2017; Ortmann and Kolaczkowska, 2018). We outline the main findings on innate immune cells in elderly and CCS below.

#### Monocytes/Macrophages

Monocyte/macrophage activation status and function are essential for appropriate immune responses against pathogens and transformed cells and for mounting the adaptive immune response. Data from human and animal studies show age-related dysfunction of the monocyte/macrophage system (Solana et al., 2012; Linehan and Fitzgerald, 2015). A detailed overview addressing the impact of ageing on monocyte/macrophage function is beyond the scope of this article and more comprehensive reviews can be found elsewhere (Shaw et al., 2010; Solana et al., 2012). Here we will highlight sporadic recent findings of monocyte/macrophage alteration seen in CCS, potentially associated with the chronic health condition development.

While the total number of monocytes seems to be unaffected by aging, (Seidler et al., 2010) proportional changes in monocyte subsets, including the relative expansion of CD16^+^ populations, have been observed in healthy males and females (20–84 yo) (Hearps et al., 2012). The CD14^+^CD16^+^ monocyte subset is also expanded in CCS (Sulicka et al., 2013) (Table 2). Since CD16^+^ monocyte subsets are characterized as significant producers of pro-inflammatory cytokines, (Hearps et al., 2012) their proportional increase in the elderly and CCS may contribute to CLGI and thus to the pathogenesis of inflammatory diseases. Moreover, significantly shorter relative telomere length has been determined in monocyte subsets from older individuals. Although information about monocyte telomere length in CCS are not available, shortened leukocyte telomere length has been reported in CCS (Song et al., 2020). Whether telomere shortening affect monocyte function remain to be elucidated. Nevertheless, shorter leukocyte telomeres have been associated with chronic health conditions in elderly and CCS (Spyridopoulos et al., 2009; Song et al., 2020). Although older individuals exhibit dysregulated inflammatory response, (Hearps et al., 2012; Metcalf et al., 2017) no data on monocyte functions, including phagocytosis or TLR stimulation, are available in CCS at the moment. This knowledge gap provides an opportunity for future research studies in the CCS field.

#### Neutrophils

Neutrophils are professional phagocytes adapted to perform rapid immune action at the site of infection or tissue damage. Even though neutrophil counts remain stable during an individual’s adulthood, (Solana et al., 2012) profound age-related changes at the functional level have been reported. In particular, neutrophils from elderly subjects exhibit reduced migration potential, phagocytosis, bactericidal activity, ROS production, and neutrophil extracellular trap formation (Wenisch et al., 2000; Butcher et al., 2001; Simell et al., 2011; Brubaker et al., 2013; Hazeldine et al., 2014; Sapey et al., 2014; Itagaki et al., 2015; Sauce et al., 2017). By contrast, evidence of increased neutrophil proteinase activity and upregulated degranulation has been observed in older adults (Sapey et al., 2014). Interestingly, in a mouse model, a higher level of neutrophil elastase was associated with a low-grade inflammatory state accompanying obesity (Talukdar et al., 2012). In CCS, a low absolute neutrophil count (neutropenia) has been reported as a consequence of intensive cancer treatment and possess a higher risk of infection (Han et al., 2009). Studies on neutrophil functionality in CCS are lacking but we can speculate that neutrophils of CCS might possess similar defects as seen in elderly. Thus, the impaired effector function of neutrophils might be involved in the increased susceptibility of some CCS to microbial infections.

#### Dendritic Cells

Dendritic cells (DCs) are professional antigen-presenting cells that can prime naive T-cell activation and effector differentiation. In general, the phenotypes and numbers of DCs are largely unaffected by aging, (Granot et al., 2017) although some studies have reported a marked decrease in plasmacytoid DCs (pDCs) (Pérez-Cabezas et al., 2007; Jing et al., 2009; Garbe et al., 2012; Splunter et al., 2019) and in CD141^+^ myeloid DCs (mDCs) upon *in vitro* stimulation with retinoic acid in healthy elderly (Agrawal et al., 2016). Since pDCs play key role in antiviral responses, their reduction in combination with mDC dysfunction may partially explain higher incidence of viral infections in elderly (Jing et al., 2009). The same mechanism might be critical for some CCS, although there are currently no studies regarding DC phenotype and function in CCS.

Concerning DC function, the following alterations have been reported in elderly individuals: decreased capacity to phagocytose antigens, (Agrawal et al., 2007) migrate, (Agrawal et al., 2007) prime naive T cells after stimulation, (Sridharan et al., 2011) respond to TLR stimulation, (Panda et al., 2010) secrete type I and III interferons (Panda et al., 2010; Qian et al., 2011; Sridharan et al., 2011). Furthermore, these cells produce increased levels of pro-inflammatory cytokines, and decreased levels of anti-inflammatory and immune-regulatory cytokines (summarized in review) (Agrawal et al., 2017). Overall, the disruption of DC functions followed by increased pro-inflammatory potential might result not only in higher risk of infections but also to CLGI and loss of tolerance. Thus, DC dysfunction likely contribute to chronic age-associated diseases in elderly and probably in CCS.

#### Natural Killer Cells

Natural killer (NK) cells are a subset of cytotoxic non-T lymphocytes that promote an early innate immune response by recognizing and killing virus-infected and tumor cells. Thus, quantitative and functional NK cell defect may lower resistance to infections and protecting effect against tumors in elderly/CCS. While the distribution of NK cell subsets seems to be altered in elderly individuals, the data are inconsistent (Pinti et al., 2016). A decrease in CD56^bright^ NK cells as a possible consequence of limited production of new NK cells, (Chidrawar et al., 2006; Le Garff-Tavernier et al., 2010) with a concomitant increase in CD56^dim^ NK cell population, has been reported in the elderly (Le Garff-Tavernier et al., 2010). Similar to T cells, CD57 expression can be used as a marker of replicative senescence in NK cells (White et al., 2014). The aging-related changes in NK cells described in the elderly population have not been studied in CCS. Nevertheless, decreased or absent NK cell cytolytic activity in 25% of patients has been reported in CCS 6 months after completion of leukemia therapy (Perkins et al., 2017). If altered NK cell function is transient or persist for years after therapy completion remains to be determined.

## Hematopoietic Stem and Progenitor Cell Aging in the Elderly and Childhood Cancer Survivors

Thus far, we have discussed how age-related alterations of individual immune cell types and their function underlie aging. However, there are also data to suggest that the age-related dysfunction of effector immune cells is in fact inherited from their progenitors. Throughout our lifespan, encounters with various stressors (e.g. allergens, viruses, bacteria, therapy of more or less severe disorders and diseases) progressively impair various cellular repair mechanisms. A consequence of inadequate repair is hematopoietic stem and progenitor cell (HSPCs) aging (Todhunter et al., 2018). Key markers of HSPC aging include higher levels of oxidative stress and DNA damage response rates, expression of p21–p53, senescence-associated β-galactosidase, and shorter telomeres (Fali et al., 2018). Additionally, the expression of genes involved in hematopoiesis, leukocyte activation, and intracellular signaling is downregulated in aged human bone marrow-derived HSPCs (Rundberg Nilsson et al., 2016).

Aged HSPCs in humans are associated with perturbed lymphopoiesis (Kuranda et al., 2011) and increased myeloid cell differentiation, (Pang et al., 2011) underlined by epigenetic changes (Beerman et al., 2013) and an age-related higher frequency of hematopoietic stem cells (HSCs) within the bone marrow (Pang et al., 2011; Farrell et al., 2014). However, data from a study on bone marrow and blood samples from patients undergoing hip replacement surgery suggests lower frequencies of HSCs in both, the bone marrow and peripheral blood in the elderly (Brusnahan et al., 2010).

Another age-related phenomenon of clonal hematopoiesis is associated with a risk of developing hematologic malignancies and an increase in specific somatic mutations in peripheral blood cells with age (Genovese et al., 2014; Jaiswal et al., 2014). These mutations have been further associated with an increased risk of myocardial infarction and coronary heart disease (Jaiswal et al., 2017). However, these mutations occur in a relatively small fraction of individuals (12.5%) as was demonstrated in the study population of Icelanders (11,262 men and women, median age of 74 years) (Zink et al., 2017). However, prevalence of these mutations increased towards 50% in subpopulation of Icelanders >85 years. Interestingly, 10 year survivorship of individuals >80 years of age seems unaffected by carrying the mutations in two of the most common genes associated to clonal hematopoiesis DNMT3A and TET2 (van den Akker et al., 2016) compared to middle-aged population study (Jaiswal et al., 2014).

HSC transplantation is a frequently used therapeutic option in childhood cancer treatment, but only few researchers have assessed its long-term impact on overall immune health in CCS. Telomere length in HSCs rapidly shortens upon transplant while replenishing the pool of blood cells. Recipients of HSC transplants have shorter telomeres in leukocytes compared with their HSC donors, (Wynn et al., 1998; Baerlocher et al., 2009) suggesting a higher turnover of recipient HSCs to ensure immune recovery. A high degree of telomere loss might occur between CD34^+^CD38^−^ and CD34^+^CD38^+^, (Vaziri et al., 1994) while more committed progenitors are more rapidly replenishing the pool of blood cells in recipients (Wynn et al., 1999). Therefore, the higher turnover rate of HSCs may predispose the recipient to an increased risk of accelerated aging of the hematopoietic compartment. This suggestion has been confirmed in serial transplantation assays *in vitro*, which showed that HSCs with increased cell cycle activity resulted in shorter telomeres, (Allsopp et al., 2001) even though HSCs exhibit some telomerase activity (Morrison et al., 1996). Moreover, HSCs after serial transplantation possess high levels of ROS, (Jang and Sharkis, 2007) which could lead to post-transplant HSCs suffering from oxidative damage to cellular components and altered metabolism and intracellular signaling (Zhang et al., 2016; Forrester et al., 2018; Milkovic et al., 2019). Although the telomere length of leukocytes in HSC transplant recipients and other markers described previously, such as high ROS and altered intracellular signaling, suggest immunosenescence in CCS, there are no studies confirming the presence of HSC aging markers and their fitness in CCS compared to the elderly. Moreover, there are no studies describing HSC aging in CCS comparing individuals with and without HSC transplantation. Even though, HSCs aging could be caused by transplantation followed by higher turnover of HSCs, there can be other likely mechanisms involved. First of all, telomerase complex has been implicated to control hematopoietic stem cell differentiation and senescence *in vitro* (Jose et al., 2018). Second of all, mesenchymal stromal cells (MSCs), which have been described to be critical for extracellular matrix production in the bone marrow, and thus facilitation of HSCs engraftment and cell fate decision, (Zhao and Liu, 2016; Krater et al., 2017), can likely present another pro-aging scenario regarding HSCs. There has been evidence, that paclitaxel (chemotherapy drug) induces senescence in MSCs *in vitro*, (Munz et al., 2018) and SASP produced by MSCs can in turn cause senescence of other epithelial cells posing a risk for HSCs upon transplant (Alessio et al., 2019). Moreover, while MSCs are being investigated to improve the engraftment of HSCs and to overcome Graft versus host disease (GvHD) in hematological transplantations, (Lee et al., 2002; Squillaro et al., 2016; Zhao and Liu, 2016; Burnham et al., 2020) concomitant immunosuppressive treatment through inhibition of Nuclear factor of activated T cells (NFAT) signaling, which is traditionally used to prevent GvHD, (Castagna et al., 2016) can induce extracellular matrix remodelling through impaired NFAT signaling in MSCs, (Tidu et al., 2021) and thus can impact the homing and function of HSCs in the bone marrow.

Overall, there are some parallels between the decline in function of naturally aged HSPCs seen in the elderly and the post-transplant phenotype and function of HSCs in CCS. We consider that there is an urgent need for long-term follow-up studies in CCS and their healthy counterparts to directly compare HSPC status and the expression of molecular aging markers. From the resulting data, we can then determine to what extent HSPC aging might be accountable for the complications and comorbidities of CCS observed later in life.

## Chronic Low-Grade Inflammation at the Crossroads of Immune Cell Dysregulation in the Elderly and Childhood Cancer Survivors

Frailty is a common feature of aging, (Lu et al., 2016; Wang et al., 2020) but it is also seen in young adult CCS (Collerton et al., 2012; Ness et al., 2013; Ness et al., 2015). This multi-system condition is closely linked with chronic inflammation (Lu et al., 2016). Interestingly, studies in the elderly, particularly those with poor physical function or frailty, have identified the presence of CLGI that is characterized by increased circulating levels of the pro-inflammatory cytokines IL-6, IFN-γ, IL-8, IL-15, CRP, and TNF-α (Bruunsgaard et al., 1999; Franceschi et al., 2000b; Zanni et al., 2003; Gangemi et al., 2005; Koelman et al., 2019). CLGI in this population, referred to as inflammaging, (Franceschi et al., 2000a) is considered a major known contributor to clinical manifestation of age-associated diseases but does not have to be the source of disease development (Fülöp et al., 2019).

The presumed sources of the inflammatory molecules contributing to inflammaging include not only immune cells but also non-immune cells, particularly adipocytes (Mau and Yung, 2018) or senescent cells from outside of the immune system (Childs et al., 2017). Moreover, cell debris, misfolded/misplaced cell molecules, referred to as damage-associated molecular patterns (DAMPs), metabolic processes, age-associated changes in the gut microbiome, and persistent viral infection can all contribute to some degree to the activation of immune cells and the ongoing chronic inflammatory response (Franceschi et al., 2017; Chen and Yung, 2019).

Several researchers have reported a partial CLGI phenotype among CCS (Table 3), characterized by high levels of IL-6, (Azanan et al., 2016; Ariffin et al., 2017; Chua et al., 2017) but not TNF- α, and accompanied by other pro-inflammatory (IL-17a, IL-2) and anti-inflammatory (IL-10) cytokines (Ariffin et al., 2017). Nevertheless, the emerging data on CLGI phenotype is still inconsistent showing also comparable levels of TNF- α, IL-6, soluble high-sensitivity C-reactive protein (hsCRP) to healthy age-matched controls (Sulicka et al., 2013). In addition, expression of the inflammatory marker soluble CRP has been reported in several CCS cohorts (Azanan et al., 2016; Ariffin et al., 2017; Chua et al., 2017). Another sign of CLGI in CCS is the presence of CMV Ig, which is also found in the elderly population, suggesting an age-related immunophenotype (Azanan et al., 2016). Furthermore, recent findings indicate that monitoring DAMPs might have prognostic or predictive value in cancer patients because their increased level signals unfavorable disease progression and survival so could serve as a potential biomarker of the level of tissue damage during/after therapy (Fucikova et al., 2015). To date, no studies on DAMPs in CCS have been published, but future work should focus on this phenomenon in CCS. After therapy, DAMPs evaluation can provide valuable information about the patients’ response to therapy and comparison of different chemotherapeutic regimens and intensity of radiotherapy between patients. In the search for early cancer development markers, further monitoring of DAMPs during regular check-ups could potentially uncover a secondary malignancy occurring frequently as late effect in CCS.

**TABLE 3:** Immune mediators associated with the senescent/immunosenescent phenotype in cohorts of CCS and the healthy elderly detected in serum/plasma.

Protein	CCS	Healthy elderly
IL-6	ALL Azanan et al. (2016), Chua et al. (2017), Sadurska et al. (2018); AML Azanan et al. (2016)	↑ Toft et al. (2002), Forsey et al. (2003), Pedersen et al. (2003), Ferrucci et al. (2005), Donato et al. (2008), Ogawa et al. (2008), Cartier et al. (2009), Brusnahan et al. (2010), Kim et al. (2011), Njemini et al. (2011), Palmeri et al. (2012), Hooten et al. (2012), Morrisette-Thomas et al. (2014), Lane-Cordova et al. (2016), Puzianowska-Kuźnicka et al. (2016), Valiathan et al. (2016), Wyczalkowska-Tomasik et al. (2016), Castañeda-Delgado et al. (2017), Erskine et al. (2017), Rusanova et al. (2018), Koelman et al. (2019), Milan-Mattos et al. (2019)
		∼ Beharka et al. (2001), Toth et al. (2005), Slusher et al. (2019)
TNF-α	Not found	↑ Bruunsgaard et al. (2000), Pedersen et al. (2003), Sandmand et al. (2003), Ogawa et al. (2008), Cartier et al. (2009), Kaszubowska et al. (2011), Njemini et al. (2011), Valiathan et al. (2016), Saldias et al. (2017), Ong et al. (2018), Koelman et al. (2019), Slusher et al. (2019)
		∼ Toth et al. (2005), Donato et al. (2008), Kim et al. (2011), Palmeri et al. (2012), Angelovich et al. (2016), Wyczalkowska-Tomasik et al. (2016), Arroyo et al. (2017), Erskine et al. (2017)
IL-2	ALL Ariffin et al. (2017)	↑ Valiathan et al. (2016), Koelman et al. (2019)
		∼ Kim et al. (2011), Palmeri et al. (2012)
IL-10	ALL Ariffin et al. (2017)	↑ Rusanova et al. (2018)
		↓ Njemini et al. (2011), Morrisette-Thomas et al. (2014)
		∼ Forsey et al. (2003), Kim et al. (2011), Palmeri et al. (2012), Erskine et al. (2017), Slusher et al. (2019)
IL-17a	ALL Ariffin et al. (2017)	↑ Li et al. (2017)
		∼ Kim et al. (2011), Palmeri et al. (2012)
hsCRP/CRP	ALL Azanan et al. (2016), Ariffin et al. (2017), Chua et al. (2017), Sadurska et al. (2018), Masopustová et al. (2018); AML Azanan et al. (2016); Hodgkin lymphoma Cepelova et al. (2019); HR-NB Vatanen et al. (2017); ALL, Hodgkin lymphoma, Non-Hodgkin lymphoma Sulicka-Grodzicka et al. (2021)	↑ Bruunsgaard et al. (2000), Ferrucci et al. (2005), McFarlin et al. (2006), Donato et al. (2008), Ogawa et al. (2008), Cartier et al. (2009), Morrisette-Thomas et al. (2014), Halper et al. (2015), Lane-Cordova et al. (2016), Puzianowska-Kuźnicka et al. (2016), Wyczalkowska-Tomasik et al. (2016), Li et al. (2017), Milan-Mattos et al. (2019)
		∼ Toth et al. (2005), Angelovich et al. (2016)
sCD163	LEU Sandmand et al. (2003)	↑ Møller et al. (2011)

The number of studies listed in the healthy elderly group suggests the clear establishment of the marker in the aging phenotype. Increases in the elderly are marked by ↑, decreases by ↓ and no age-based difference is marked ∼. Abbreviations: ALL, acute lymphoblastic leukemia; AML, acute myeloid leukemia; HR-NB, high risk neuroblastoma; LEU, leukemia.

While the effects of different therapies might have distinct outcomes in terms of changes to the cellular immune compartment, this convergence of immune dysregulation into CLGI might enable blood inflammatory markers to be used as a relatively simple and straightforward approach to define the levels of damage in individual CCS. While the effect of CLGI in CCS has not been specifically addressed, CLGI is likely to be linked to the risk level for various comorbidities, as evidenced by the number of comorbidities and CLGI markers in the elderly. Therefore, CLGI might present a common ground not only for individuals with chronic inflammatory disorders and elderly, but also for CCS. Thus, CCS with significant evidence of CLGI could be potentially stratified for more intense monitoring and/or early intervention strategies.

## Immunosenescence-Targeted Interventions

In CCS, chemotherapy, radiotherapy, and HSC transplants have all been associated with adverse effects on the immune system through telomere shortening, phenotype/epigenetic changes, and cytokine production (Li et al., 2012; Azanan et al., 2016; Ariffin et al., 2017; Vatanen et al., 2017; Daniel et al., 2018). While therapeutics aiming to restore optimal function to the ailing immune system in CCS have yet to be developed, several groups have explored possible strategies to reduce the impact of immunosenescence in the elderly. These findings have been nicely compiled in many review articles on the topic of reversing or retarding aging, (Fulop et al., 2017; Duggal, 2018; Aiello et al., 2019; Weyh et al., 2020) and strategies, such as diet, exercise and pharmacologic therapies are proposed to remediate cancer treatment-related aging of CCS (Guida et al., 2021).

A simple and non-invasive strategy to combat immunosenescence and CLGI is to follow an active lifestyle. Exercise reduces the proportion of CD14^+^CD16^+^ monocytes and TNF-α production upon stimuli (Timmerman et al., 2008) and improves the migration of neutrophils towards IL-8 (Bartlett et al., 2016). Exercise has a positive effect on the adaptive immune system. As it increases the frequency of naive T cells and recent thymic emigrants, and lowers T-helper 17 cell polarization in those who exercise compared with less active counterparts (Duggal et al., 2018). Nevertheless, the proportion of CD28^−^CD57^+^ CD8 T cells seems unaffected. In general, it has been suggested that exercise induces a systemic anti-inflammatory response in the organism by initially IL-6 and later IL-1Ra and IL-10 expression (Petersen and Pedersen, 1985).

An interesting review by Leal et al. (2018) highlights the involvement of adipokines and myokines in the body’s response to exercise (Steensberg et al., 2003; Fischer et al., 2007; Miyamoto-Mikami et al., 2015). Nowadays, a clinical trial has been established to determine the type of exercise beneficial to the elderly (NCT04534049). The observation of the whole organism response is important, in particular, because of the comorbidities (e.g. diabetes mellitus, metabolic diseases) that are often observed in CCS (Table 1). Thus, regular exercise could also form a preventative measure.

Potential therapeutic approaches targeting the already existing immunosenescent phenotype through metabolic pathways could involve AMPK activators, mechanistic target of rapamycin (mTOR) inhibitors, and caloric restriction (Hortova-Kohoutkova et al., 2021). AMPK activators, mTOR inhibitors, and caloric restriction are all associated with increased life and/or health span (Harrison et al., 2009; Martin-Montalvo et al., 2013; Pifferi et al., 2019). Nevertheless, there are still major knowledge gaps in the crosstalk between AMPK and mTOR related to senescence and aging on the whole body and cell type/subset, as AMPK drives T-cell senescence through p38 kinase (Lanna et al., 2014) and mTOR complex 1 is involved in autophagy induction of CD8^+^CD28^+^ T cells but not CD8^+^CD28^−^ T cells via the T-cell receptor (Arnold et al., 2014). Regarding the mechanisms underlying the benefits of caloric restriction, AMPK and sirtuin 1 (SIRT1), and NAD^+^-dependent deacetylase seem to mediate the caloric restriction health benefits while triggering autophagy (Cohen et al., 2004; Lee, 2019). Sirtuins might also have protective roles in age-related disease development (Lee, 2019). For example, resveratrol, a SIRT1 activator, has anti-inflammatory effects, and can suppress TLR signaling, reduce pro-inflammatory gene expression, (Malaguarnera, 2019) and decrease age-related changes of CD4 and CD8 T lymphocytes in aged mice (Wong et al., 2011). Although caloric restriction has proven to have beneficial effects on immune cell phenotypes distribution, it may conversely increase susceptibility to infection as has been shown in aged mice (Gardner, 2005; Goldberg et al., 2015). Thus, balancing a proper diet-related strategies or interventions in the AMPK-mTOR pathways have to be in concordance with the organismal health. Inappropriate diet in individuals with obesity and/or metabolic syndrome may increase a risk of infection (Hortova-Kohoutkova et al., 2021).

Another important anti-aging strategy aims to rejuvenate the thymus in elderly (Thomas et al., 2020). The data available on thymus regeneration are so far limited to animal models; although, increased serum levels of IL-7, a thymoprotective cytokine, has been described in trained elderly cyclists (Duggal et al., 2018). Data from an early study showed that IL-7 treatment in the form of a fusion protein restored thymic cellularity and architecture, (Henson et al., 2005) producing additional *de novo* T cells and increasing thymic output. Another thymic regeneration strategy involves reprogramming fibroblasts with forkhead box N1 (FOXN1) to form thymic epithelial cells and create a functional thymic stroma, (Bredenkamp et al., 2014a) or gene therapy in the form of inducible FOXN1 expression (Bredenkamp et al., 2014b). A combination of the AMPK activator (metformin) and other active substances with the focus on immunosenescence and epigenetic markers in elderly participants is currently used in an ongoing phase 2 clinical trial on thymus regeneration (NCT04375657).

A promising therapeutic approach centers on gut microbiota manipulation. Loss of microbiota diversity is associated with aging and has also been observed in CCS (Table 1). Probiotic supplements are already widely used by cancer patients with questionnaire-based positive results (Ciernikova et al., 2017; Nazir et al., 2018). Further studies on the long-term use of these supplements that evaluate the progression of comorbidities and immunosenescence are needed. Studies on the gradual change of microbiome diversity with aging (Xu et al., 2019) and in CCS (Chua et al., 2017) can serve to optimize probiotic composition.

Some strategies to overcome immunosenescence, namely exercise and probiotic supplements, can be relatively smoothly implemented in CCS post-therapy regimens, and studies on their quality of life and immunosenescence markers are much needed. These strategies can prove to be helpful rather than harmful by off-target effects in both groups, CCS and elderly. Moreover, implementation of exercise in young cancer patients or CCS could have many additional benefits including a motivation to meet with their peers, retrieve emotional and social stability (Gallicchio et al., 2008; Effinger et al., 2019) and thus overall higher quality of life. Unlike CCS, the elderly might have many physical limitations already unable to perform exercise such as longer-distance walking and thus performing exercise presents a rather preventative strategy for exercise-habituated aging individuals. The approved metabolic manipulation strategies can be used in CCS as well as in elderly when advised by and closely examined its suitability to the overall health status by their general practitioners/specialists. Unlike some above-mentioned strategies, thymus regeneration strategies are still in the process of development and thus not approved yet. Nevertheless, this strategy might be more beneficial to the elderly rather than CCS depending on the rate of thymic involution and thymic output. Lastly, so far unapproved strategies based on interfering with metabolic and signaling pathways have to be carefully studied in mouse models and in clinical trials because of possible off-target effects.

## Childhood Cancer Survivors Risk Stratification Strategies

Specialised life-long follow-up care programmes for CCS are gradually being established across the world’s healthcare systems (Jacobs and Shulman, 2017; van Kalsbeek et al., 2021). In order to provide personalised, effective and also cost-effective surveillance of late effects, evidence-based strategies and models are sought to stratify CCS by risk of individual late effects. However, current risk-stratification schemes (Wallace et al., 2001; Edgar et al., 2013; Frobisher et al., 2017) are largely based on intensity of treatment, especially cumulative doses of chemotherapy and type and intensity of radiotherapy, and do not suffice to explain many variations in individual responses to treatment.

**FIGURE 3:**
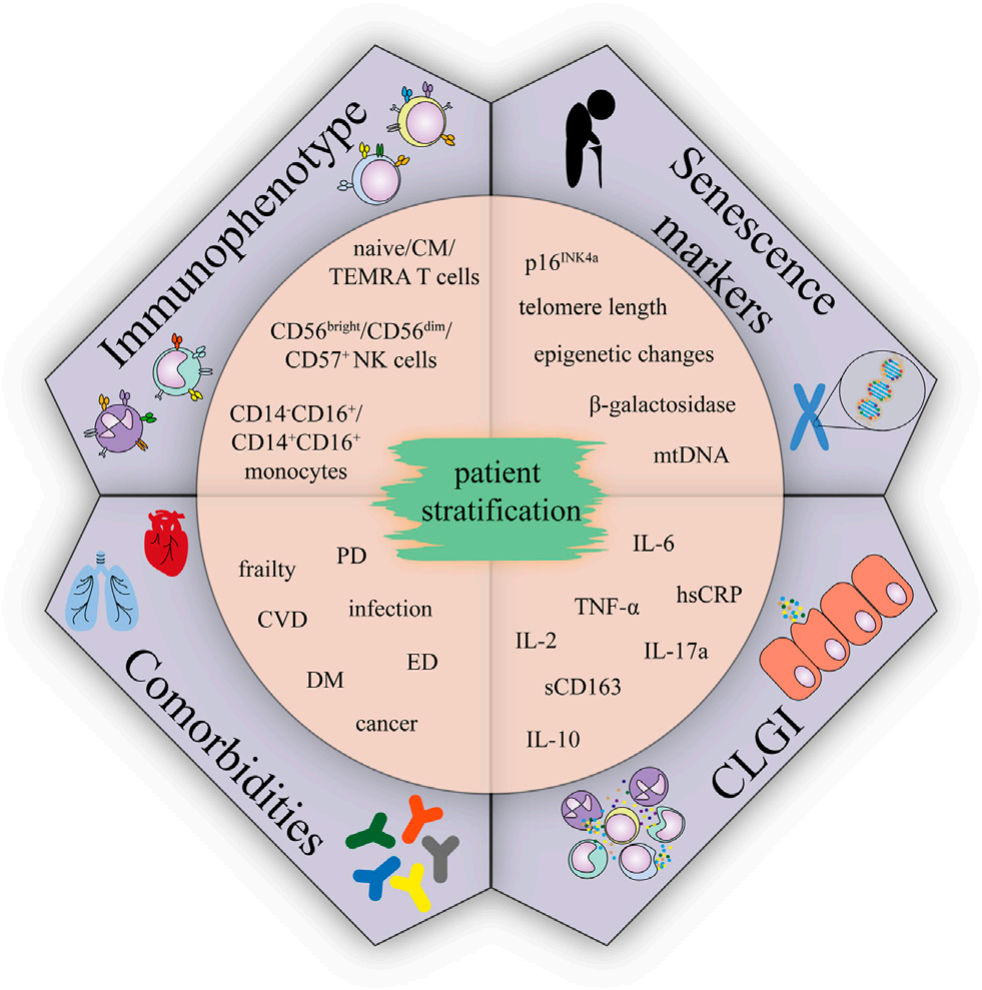
Immunophenotype, comorbidities, CLGI, and senescence markers suggested for CCS patient stratification.; Abbreviations: CM, central memory; TEMRA, terminally differentiated effector memory T cells re-expressing CD45RA; CVD, cardiovascular disease; DM, diabetes mellitus; ED, endocrinal disease; PD, pulmonary disease.

Given the CLGI is the condition contributing to aetilogy of many late effects, and concurrently is associated with immunosenescence, we suppose that accelerating aging phenotype of CCS might be accompanied by immunosenescence. Understanding the relationship between age-related immunosenescence and immune features of CCS may allow the identification of new markers. Combination of conventional factors (e.g cardiovascular – hypertension, diabetes, dyslipidemia) (Armstrong et al., 2013; Chow et al., 2014) and newly identified markers may improve currently existing risk stratification models. Such a classification of CCS into groups can eventually become the subjects of different prevention regimens/programs. Based on the reviewed literature, several candidate markers could be promising tools to screen CCS cohorts: 1) analysis of serum/plasma for major markers of the senescent phenotype (TNF-α, IL-6, hsCRP, IL-10, IL-2, IL-17A and sCD163), 2) analysis of cellular senescence in the subsets of peripheral blood cells (T cells, NK cells, and monocytes), 3) senescence markers (telomere length, p16^INK4a^ expression, β-galactosidase, epigenetic changes (T cells and other peripheral blood mononuclear cells)) and 4) presence of health complications (Figure 3). Detection of these markers in CCS cohorts is likely to correlate with, and may even predict, the risk of developing serious typical age-related polymorbidities and frailty and would be a useful first step towards a more detailed understanding of the mechanistic link between comorbidities in CCS, immune phenotypes, and premature aging.

One notable effect of cancer therapies, including chemotherapy, (Li et al., 2012) radiotherapy, and bone marrow transplantation, (Schröder et al., 2001) is the disruption of telomere homeostasis. Furthermore, telomere attrition is promoted by an inflammatory environment (Jose et al., 2017) and has been described as among the first systemic hallmarks of aging in the elderly, (Woo et al., 2014) accelerated aging in chronic disorders, (Zhao et al., 2013; Haycock et al., 2014) and aging in CCS (Ness et al., 2018). Significantly shorter leukocyte telomere length has been reported among CCS exposed to various cancer treatment in comparison to non-cancer control (Song et al., 2020) and has been associated with prevalence of chronic health conditions in CCS. Interestingly, favourable health behaviours have been associated with longer leukocyte telomere length of younger CCS. Song et al. (2020) suggests telomere length as a promising aging biomarker which may be involved in strategies for health promotion and disease prevention in CCS particularly with regard to aging-related chronic health conditions.

Here, we hypothesize that the crosstalk of telomere control mechanisms and chronic inflammatory disorders is another crucial factor in the development of accelerated senescence in CCS. The fact that telomere shortening is induced by anticancer therapy, and at the same time clearly correlates as an additional risk for further development of cancer makes telomere length assessment a strong candidate for stratification of CCS at different risks of further adverse effects.

Several systemic markers correlate with the aging process (Herranz and Gil, 2018; Wiley and Campisi, 2016) (Table 4). The markers observed in peripheral blood cells in CCS include apart from telomere shortening also expression of p16^INK4a^ and epigenetic changes. Moreover, p16^INK4a^ expression in T cells correlates with intensity of chemotherapy and frailty in the CCS, and thus cellular senescence has been proposed to be associated with premature aging in CCS (Smitherman et al., 2020). Another established aging marker accessible from plasma, circulating cell-free mitochondrial DNA, has not yet been studied in CCS and neither has been β-galactosidase, a well-known marker for senescence (Dimri et al., 1995). Interestingly, β-galactosidase has been reported in only one study recently in peripheral blood cells (terminally differentiated CD8^+^ T cells specifically) of healthy elderly, (Martínez-Zamudio et al., 2021) and a study by Spazzafumo et al. (2017) reports increased β-galactosidase activity in plasma of elders (Spazzafumo et al., 2017). Nevertheless, none of those parameters has ultimate informative value without the context of many other parameters (Casella et al., 2019).

**TABLE 4:** Systemic markers of senescence/aging in peripheral blood cells and plasma.

Marker	CCS	Healthy elderly
Telomere length	ALL Ariffin et al. (2017); various cancer types Song et al. (2020), Gramatges et al. (2014), Franco et al. (2003), NHL Lee et al. (2003); HR-NB Vatanen et al. (2017)	Bischoff et al. (2005), Sanders et al. (2009), Haapanen et al. (2018), Montiel Rojas et al. (2018), Kuo et al. (2019)
p16^INK4a^	Various cancer types Smitherman et al. (2020)	Liu et al. (2009), Song et al. (2010), Kao et al. (2016)
Epigenetic changes	Various cancer types Daniel et al. (2018)	Bell et al. (2012), Johnson and Conneely (2019)
Circulating mtDNA, cf-DNA	Not found	Jylhävä et al. (2013), Pinti et al. (2014), Teo et al. (2019)
β-galactosidase	Not found	Spazzafumo et al. (2017), Martínez-Zamudio et al. (2021)

Abbreviations: ALL, acute lymphoblastic leukemia; Cf-DNA, cell-free DNA; HR-NB, high-risk neuroblastoma; mtDNA, mitochondrial DNA; NHL, non-Hodgkin’s lymphoma.

In summary, further analysis of candidate markers shared between elderly and CCS might help to develop a risk-stratification profiles for identification of high-risk (intense monitoring and early intervention) and low risk (reduced monitoring) subpopulation groups. The appropriate patient stratification would enable increased screening and pro-active early clinical management of these patients.

## Future Perspectives

The parallels between immune senescence in the elderly and immune alterations seen in CCS are, in some cases, quite notable; however, the definition of altered immune states in CCS is incomplete. The specific effects of different cancer treatments on the young immune system are yet to be fully understood, and long-term follow-up studies are overall lacking. Moreover, another hurdle is that the data available for CCS immune cell phenotype and CLGI consist almost exclusively of leukemia survivors, while the comorbidities cover multiple cancer types—leukemias, lymphomas, brain and nervous system tumors etc. The question is, whether the studies were done on leukemia survivors on purpose due to the highest incidence reaching up to 25% of all pediatric neoplasms, (Howlader et al., 2020) and thus achieving a phenotype assessment in a relatively homogeneous group of CCS, or the immune senescence and CLGI occur predominantly in this type of pediatric cancer due to its specific therapeutic regimen including antracyclines and alkylating agents (in 73% of CCS), and radiotherapy (in 52% of CCS) (Azanan et al., 2016; Ariffin et al., 2017). We recently showed that high-risk neuroblastoma CCS exhibited transient signs of an immunosenescent-like phenotype, but this was resolved after 5 years, (Lázničková et al., 2020) illustrating the dynamic nature of immune recovery from cancer treatment and the need for larger longitudinal studies across tumor types. Although more than two-thirds of CCS experience notable ill health in the medium term, one-third do not. As yet, it is unclear whether the immune systems of those patients in better health are more like those of healthy age-matched peers or whether other factors are responsible for the emergence of typical age-related conditions in these young adults. Genetic factors, including those for metabolic diseases, that predispose to obesity, hypertension or diabetes mellitus have not provided sufficient predictive value in CCS, presumably due to low patient numbers in genetic studies and lack of control cohorts (Wilson et al., 2015; Clemens et al., 2018). So far, only one study showed association of leptin receptor-encoding gene polymorphism and obesity in irradiated female CCS (Ross et al., 2004).

Another possible explanation for the differences in the immune features between CCS and healthy peers is that immune alterations may pre-dated treatment, or even the cancer itself. Therefore, future research should be directed to investigate mechanisms behind these alterations resulting in identification of immune parameters allowing prognosis prediction. One such predictive pre-treatment immune cell characteristic has been already attributed to the neutrophil-to-lymphocyte ratio in pediatric patients with solid brain tumors who have elevated neutrophils and decreased lymphocyte counts compared to children with unrelated diagnoses prior to surgery (Yalon et al., 2019). These data remain to be validated for other pediatric cancers; however, data has been collected for various adult cancers (Howard et al., 2019). Nevertheless, it remains important to understand the present state of the immune system status in CCS due to the high number of CCS across the globe and the tight association of the immune system with the development of comorbidities, including possible relapses or secondary cancers.

In summary, we consider that the late adverse effects in CCS are similar to the immunosenescence-related age-associated morbidities observed in the elderly. Our belief is that immunosenescent phenotyping could provide crucial information for patients struggling with the therapy-induced morbidities that occur in CCS. This link is especially relevant considering the growing research emphasis on ways to ameliorate immunosenescence in the elderly. If relevant parallels exist between these two patient groups, then similar strategies could markedly benefit CCS. The increasing numbers of CCS are an undisputable testament to one of the biggest successes of oncological research in previous decades. The challenge moving forward is to ensure that cancer cures are not necessarily accompanied by a future of chronic conditions with the potential to limit both quantity and quality of life.

## Abbreviations

ABC, Age-associated B cell; AMPK, AMP-activated protein kinase; CCS, Childhood cancer survivor; CLGI, Chronic low-grade inflammation; CMV, Cytomegalovirus; DAMPs, Damage-associated molecular patterns; DC, Dendritic cell; FOXN1, Forkhead box N1; hsCRP, High-sensitivity C-reactive protein; HSC, Hematopoietic stem cell; HSPC, Hematopoietic stem and progenitor cell; KLRG-1, Killer cell lectin-like receptor subfamily G member 1; LM, Late/exhausted memory B cell; NK, Natural killer cell; mDCs, Myeloid dendritic cells; mTOR, Mechanistic target of rapamycin; pDCs, Plasmacytoid dendritic cells; ROS, Reactive oxygen species; SASP, Senescence-associated secretory phenotype; SIRT1, Sirtuin 1; TLR, Toll-like receptor.
